# Balancing different views in the contexts of voluntary assisted dying, safe access zones around reproductive health clinics, and the proposed *Health Legislation Amendment (Conscientious Objection) Bill*

**DOI:** 10.1007/s40592-025-00227-4

**Published:** 2025-04-13

**Authors:** Fiona Patten

**Affiliations:** Australian Senate candidate for the Legalise Cannabis Party, Melbourne, Australia

**Keywords:** Religious pluralism, Values, Voluntary assisted dying, Abortion, Safe access zones, Conscientious objection

## Abstract

The following text is the de-identified and edited transcript of an invited presentation by Fiona Patten, who was a member of the Victorian Legislative Council between 2014 and 2022, and leader of the Reason Australia Party. Fiona’s presentation addressed the topic of ‘Balancing different views in the contexts of voluntary assisted dying, Safe Access Zones around reproductive health clinics, and the proposed *Health Legislation Amendment (Conscientious Objection) Bill*.’ Fiona’s presentation formed part of the Conference on Accommodating Plural Values in Healthcare and Healthcare Policy, which was held in Melbourne, Australia, on Monday, October 30, 2023. This conference was a key output of the Australian Research Council Discovery Project grant DP190101597, ‘Religion, pluralism, and healthcare practice: A philosophical assessment’. Fiona’s presentation was introduced by the project’s principal investigator and Deputy Director of Monash Bioethics Centre, Professor Justin Oakley.

## Foreword - Introduction to presentation by Professor Justin Oakley (Principal Investigator of the Australian Research Council Discovery Project DP190101597, ‘Religion, pluralism, and healthcare practice: A philosophical assessment’, and Deputy Director, Monash Bioethics Centre, Monash University)

[Justin Oakley] Fiona Patten and Margaret O’Connor are our speakers for the next part of this morning, and I’m really excited to hear what they have to say about their involvement in the development of Victoria’s voluntary assisted dying laws.

I’ll tell you a bit about Fiona. Fiona was a member of the Victorian Parliament from 2014 to 2022 and initiated and delivered many reforms, including voluntary assisted dying, abortion law reform, spent convictions, the decriminalisation of sex work, and drug law reform. While in Parliament, Fiona raised many other issues, including legislation to end corporate conscientious objection in public religious hospitals. Fiona was the first independent chair of the Legal and Social Issues Standing Committee, which delivered landmark reports into homelessness and the criminal justice system. Since leaving Parliament, Fiona has continued to work on social reform issues, including women’s reproductive health, criminal justice reform, the regulation of adult use of cannabis and a greater separation of church and state. In 2018, she published her autobiography, *Sex, Drugs and the Electoral Roll*, and she is the former leader of the Reason Party. Fiona’s topic is balancing different views in the context of voluntary assisted dying, safe access zones around reproductive health clinics and the proposed *Health Legislation Amendment (Conscientious Objection) Bill*. Over to you, Fiona.

## Transcript of Fiona Patten’s presentation

[Fiona Patten] Thanks Justin and good morning everyone. And good morning everyone on Zoom, I’ll hope you’ll have lots of questions. I’d like to just acknowledge that we are on the land of the Wurundjeri people of the Kulin nation and I pay my respects to any elders who are here today and those emerging. And always recognise that whenever we’re talking about healthcare, in whatever aspect we are, our Aboriginal brothers and sisters are the most disadvantaged in our healthcare settings. Pretty much without fail – or *with* fail.

So in thinking about the balancing of different views, I’m going to speak to this subject as a former politician, and as someone who was in Parliament when we debated voluntary assisted dying—in fact, I had played a longer role in that—safe access zones around abortion clinics, the decriminalisation of sex work and changes to the way publicly funded religious hospitals could use the sense of corporate conscientious objection.

And in thinking about this over the weekend, I was trying to work out—how do I balance this? To be honest, in Parliament it seemed to be constantly a battle. We seemed to be constantly fighting differing views, and it was around concession making. I don’t think it was ever really around consensus, we made concessions to groups of people, and for a range of reasons. But fundamentally, I found that it was just the privilege of religious organisations and the way that they are still pervading our parliaments and governments and the special place that they play in government, in Parliament, and therefore also in policy making.

There was an article today in the paper about how, every morning, the Lord’s Prayer is recited in Parliament. Now that sets the day and we’ve fought to have that changed, and we’re seeing local governments change that. But still, a large percentage of our state, federal and local governments are required to recite a Christian prayer before they start the work of the day. So that kind of sets the place for it. The funding for Catholic education is treated entirely differently to the funding for other schools, for other public schools, they have an *extraordinary* amount of freedom. They’re given one bucket of money and then they can decide how it’s spent on students. Religious organisations are still treated very differently, and MPs, members of parliament, still fear their wrath. They still fear it.

And I *saw* this pressure coming to bear in the Parliament, we saw the impact of the groups of religious organisations who came in to try and counter and try and stop the passage of the voluntary assisted legislation here. We’ve seen it in every subject that I’ve mentioned, whether it was on safe access zones, whether it was on sex work, whether it was on religious hospitals and obviously of course things like changing the Lord’s prayer as an opening to Parliament for something more secular, or something that reflected the diversity of our community today. And Margaret’s going to speak a lot about the voluntary assisted dying legislation that we have here.

But we’ve seen this debate not just in Victoria, we’ve seen it going on for decades. We have to remember that the first assisted dying legislation in Australia was 1996 and it was in the Northern Territory. It was *quickly* overruled by Minister Kevin Andrews on religious grounds, effectively. He may have tried to couch it in something else, but it was his deep religiosity that enabled him to successfully [overrule this legislation], and the religiosity of his colleagues, in the John Howard Government of that time. So we saw that overruling in the Northern Territory. We have seen attempts for voluntary assisted dying to be debated in the Victorian Parliament for decades. I [joined parliament] in 2014 and had made a commitment that the first thing I would do [is begin an inquiry into assisted dying], and remembering I was elected as the Australian Sex Party. So death was not what people were expecting me to start my term with, but it was absolutely the first thing that I put a motion on the notice paper to do. And then that started the end-of-life choices inquiry that we conducted here in Victoria that as we know resulted in legislation and as we’ve now seen has resulted in legislation across the country. I don’t think any of us thought that when the legislation was passed in Victoria in 2017 that within five years we would see every state in Australia adopt, change the legislation around assisted dying. And see Kevin Andrews’ legislation that stopped the Northern Territory from introducing assisted dying being overturned, opening the door for Northern Territory and the Australian Capital Territory to also start this.

And one of the things that we found with the inquiry into assisted dying was that there were organisations that opposed the passage of assisted dying. I’d have to say that the vast majority were on religious grounds. There were people like Palliative Care Victoria who certainly would argue that it wasn’t on religious grounds. However, I would dispute that. And I would dispute that when I looked at the membership of Palliative Care Victoria. Today in Victoria palliative care is still largely provided by religious organisations. And we know that this is causing some significant suffering for people at end of life who are not able to get the treatment that they want.

Now thinking about again balancing the idea of plural views around voluntary assisted dying. We saw that in all of the polling that was done around that time, it did not matter whether you held religious views or not, you were largely supportive of assisted dying. I think in Victoria 80 to 85% of Victorians [polled] believed that the legislation should pass, that people should be able to have greater decision making at the end of life. No doubt those who had very strong views against this, they would have been grounded in their religious views, but we saw amendment after amendment coming through the Parliament in the 70 odd hours of debate around this legislation. We saw MPs being threatened that they would lose their preselection if they supported this legislation. I actually know *exactly* the electorates they are, I won’t say them, but these were where the parties in those places were dominated by very conservative organisations where there was a very strong component of religious organisations who made up the membership of those political parties. So we had Members of Parliament being threatened that they would lose their preselection, that they would lose their funding in the 2018 election. And I know of two MPs who did lose their funding from their party. And coincidence or not, both of them supported assisted dying and neither of them were reelected in 2018. And one of them lost their preselection in an upper house seat to a very conservative member of their party. So we saw that.

Hour after hour, day after day, we were debating this bill. And we knew at the end that we actually could pass this bill, we knew that we had the numbers, but there were still people in the Parliament holding vigil, trying to get any moment to convince any one, one or two politicians, to change their view. So we started to see the concession making. So we saw things like, well, voluntary assisted dying had to be on your death certificate. Why? These are people at the end of their life. They are dying from – whether it’s cancer, motor neurone disease, whatever, you know, pulmonary disease – they are dying from that. That is what is going to kill them. But there was a concession that if you accessed voluntary assisted dying, that had to go on your death certificate. Now I think there was this notion that this would dissuade people, if they knew that this was going to happen.

There was also the element where doctors did not have to refer patients. So we know in abortion legislation a doctor can have a conscientious objection, but they still must refer their patient. We know that they don’t. We know that in many circumstances patients are not being referred to care or referred to another doctor. But in this case, we made this concession that doctors would not have to even have the conversation, and if a patient asked them, they could refuse to enter into any conversation about it.

And then probably the most insidious in this is the notion of corporate conscientious objection, that any facility can refuse a patient or a resident access to assisted dying for no reason except that the organisation that funds that facility does not support assisted dying. It completely ignores that notion of patient- or person-centered care, where it should be the patient’s values and wishes that should be most respected. And we’re seeing the consequences of this today. We’re seeing story after story where people have had to work *so hard* to access assisted dying. They have had to go through so much suffering to access assisted dying. To end their suffering they have had to go through more suffering. And just over the weekend in thinking about today, I was looking at the various articles that have been coming forward. I hope that we can fix some of this in the review of the legislation, but I don’t hold much hope. I think this even though the vast majority of Victorians support this legislation, they believe that people should have the right to have greater agency on how they die and when they die, when they are terminally ill. We will not see many changes to the legislation and it still will be a *battlefield* for many people to be able to access it. A *battlefield* when they have less than six months to live, when we know they’re dying. But, anyway, I know Margaret will speak more about this.

I also wanted to touch on safe access zones around abortion clinics. So basically we’ve had this issue for decades, not just in Victoria but around the country and around the world, where religious organisations come and harass, insult, pray for people seeking abortions. They stand outside clinics handing out photos or trying to dissuade people from going in to have an abortion. They harass the staff who work in those clinics. As we know in Victoria some decades ago a security guard was killed by a person who had a very strong view around abortion clinics and around abortion.

So in putting up this legislation, I thought it was very sensible. Feel free to protest about abortion. Feel free to have your own views. You just can’t impose them on someone going into a health facility. And we set a bubble, said anyone walking into that health facility can have a little bit of privacy and a little bit of peace, in the last 100 or 150 m before them going in there. Feel free to protest at Parliament. Feel free to protest wherever you like, up Collins Street. And only just a couple of weeks ago they were protesting up Collins Street. But not against people at a very difficult time in their life, making a very difficult decision, should you be allowed to harass them. Again, there was 64 h of debate about the free speech of religious organisations, and that this was not, they weren’t harassing, they were just trying to put their views across to individuals going in to get an abortion. 72 h of debate, lots and lots and lots of debate. We finished at 3:00 AM one day and then we went back the next day and finished at about 1:00 AM the next day. And every Tuesday they protest outside Parliament. But again, this was the challenge, and it was difficult. And there was a reluctance from both the Labor Party and the Liberal Party to support this legislation initially because of their fear of the response from religious organisations who had been very loud. Now in that one, also in assisted dying, we got so many submissions from people who had to remain anonymous because they worked for religious organisations. They were totally supportive of assisted dying. They told some of the most tragic stories.

And the same when we got to conscientious objection in public hospitals, where they were telling these most *gruelling* stories of where they had a patient who needed their help, but they couldn’t help them because of the conscientious objection position of the corporation that they were employed by. And we still face that today. And I hope that that you in this room will solve this problem today, possibly not today, but I hope you will find us a pathway.

I also raised the issue of conscientious objection in public hospitals, not just in Victoria, but nationally. In Melbourne we have two obstetrics hospitals. One is Hospital A, and we’ve got Hospital B. If you’re zoned to Hospital A, you cannot get contraception after your pregnancy, after your birth, you cannot get a tubal ligation even if you’re having a Caesarean. And certainly if you need a termination or an abortion as part of your care, well, you need to find somewhere else, and obviously in those circumstances you need to find it very quickly. We had patients who had turned up telling us their story of turning up to the emergency at Hospital C, obviously in need of a termination, to be refused. And this was due to [the organisation’s] conscientious objection.

We tried to raise this. I tried to say that this suppresses the conscience of those doctors and healthcare practitioners who wanted to do the right thing, who wanted to do what their patient not only wanted but needed and that the corporate conscientious objection actually denied the conscience of those who worked in those organisations. The fact that these were publicly funded hospitals that could say “you must be discharged from our facility if you want to access a legal medical treatment”, whether this was in the case of assisted dying, abortion, or contraception.

It was *remarkable* how quickly both sides of the Parliament were quick to say no. “Oh no, this is not a problem in Victoria.” It *is* a problem. Like if you look at where you try and get an abortion in Victoria there’s a real desert out there, particularly if you’re trying to get an abortion after 12 to 14 weeks. You would not want to live in Western Victoria. You would not want to live in Northern Victoria. You know, the hangover of RU-486. I don’t know if people in the room, some of you were not born yet when we had a debate about medical abortion and about bringing in a product called RU-486, I think it’s called MS-2 step now. But we’re still catching up because that was prohibited for years. That was a deal that the then Catholic Health Minister Tony Abbott did with the then Catholic senator from Tasmania, Senator Harradine, who held the balance of power.

Now as a result of that, we are still catching up. We are still one of the few countries where it is difficult to get a medical termination. You are more likely to be offered a surgical one in Australia than a far less invasive medical termination. Now that’s changing, but it’s changing slowly, and people are still scared.

We had the federal committee who looked at the dearth of women’s reproductive health services around the country and the fact that a large number of publicly funded hospitals did not provide reproductive health services, did not provide abortions, and this was particularly important out in regional areas. And the Labor government, progressive ministers in this area, the committee could only get to the point where they would recommend that publicly funded hospitals were equipped to possibly provide these services. They could not get to the point where they said that publicly funded hospitals *must* provide these services if they’re needed. So we still have a *long* way to go on this, and these are publicly funded organisations and publicly funded hospitals.

So I know the previous speakers recently touched on the topic of transgender health in young people, and we’re seeing that play out in the parliaments at the moment and we’re seeing the most *hurtful* conversations. We had a protest up in Parliament, which was largely by very conservative religious people campaigning and protesting about transgender people. Now, fine, I accept that. The Nazi sympathisers came. The *vilification* of transgender people that was being spouted at that protest and at subsequent protests, that was then reported on, and our response was to ban the Nazi salute? It was not to address the vilification of transgender people, people whose mental health tends to be *so* much worse than the general population, whose chances of suiciding is so much greater than the rest of the population. And again, this was, I think, our concessions to religious organisations, our *concessions* to those alternative views that are not, in my mind, the views of the vast majority of the population. And we seem to be constantly making concessions to organisations to the disadvantage of the majority of the community. Thank you.

## Data Availability

No datasets were generated or analysed during the current study.

